# Comparative Genomics and Characterization of the Late Promoter *p*R’ from Shiga Toxin Prophages in *Escherichia coli*

**DOI:** 10.3390/v10110595

**Published:** 2018-10-31

**Authors:** Ling Xiao Zhang, David J. Simpson, Lynn M. McMullen, Michael G. Gänzle

**Affiliations:** Department of Agriculture, Food and Nutritional Science, University of Alberta, Edmonton, AB T6G 2P5, Canada; lzhang11@ualberta.ca (L.X.Z.); lmcmulle@ualberta.ca (L.M.M.); mgaenzle@ualberta.ca (M.G.G.)

**Keywords:** Shiga toxin prophage, genomic characterization, flow cytometry, microscope, phage induction efficiency, sequence diversity

## Abstract

Shiga-toxin producing *Escherichia coli* (STEC) causes human illness ranging from mild diarrhea to death. The bacteriophage encoded *stx* genes are located in the late transcription region, downstream of the antiterminator Q. The transcription of the *stx* genes is directly under the control of the late promoter *p*R’, thus the sequence diversity of the region between *Q* and *stx*, here termed the pR’ region, may affect Stx toxin production. Here, we compared the gene structure of the *p*R’ region and the *stx* subtypes of nineteen STECs. The sequence alignment and phylogenetic analysis suggested that the *p*R’ region tends to be more heterogeneous than the promoter itself, even if the prophages harbor the same *stx* subtype. Furthermore, we established and validated transcriptional fusions of the *p*R’ region to the DsRed reporter gene using mitomycin C (MMC) induction. Finally, these constructs were transformed into native and non-native strains and examined with flow cytometry. The results showed that induction levels changed when *p*R’ regions were placed under different regulatory systems. Moreover, not every *stx* gene could be induced in its native host bacteria. In addition to the functional genes, the diversity of the *p*R’ region plays an important role in determining the level of toxin induction.

## 1. Introduction

Bacteriophages shape the genome of their prey through horizontal gene transfer, often transferring genes that provide an evolutionary benefit for both the bacterial host and the prophage. There are several examples of this phenomenon in *Escherichia coli* including phages that transfer genes into *E. coli* that confer virulence, or improve its ability to survive environmental stress [1,2,3,4]. One such group of genes are the *stx* genes that make *E. coli* toxic to some protist predators, but also convert commensal *E. coli* to human pathogens [5,6,7,8].

Shiga toxin-producing *E. coli* (STEC) cause diarrheal disease [9]. A subpopulation of STEC, enterohemorrhagic *E. coli* (EHEC), combines Stx production with adhesion to the intestinal mucosa. EHEC infections often cause fatal complications such as hemorrhagic colitis (HC) and hemolytic uremic syndrome (HUS), which can be fatal [10]. EHEC derives adhesion factors from the locus of enterocyte effacement (*eae*) of enteropathogenic *E. coli*. *E. coli* O104:H4, an emerging EHEC, caused several outbreaks in Europe from 2009 to 2011 [11,12]. *E. coli* O104:H4 combines adhesion factors of enteroaggregative *E. coli*, which produce attaching and effacing (A/E) lesions with Shiga toxin production [13]. The defining virulence factor of STEC, Shiga toxin (Stx) [14,15], inhibits protein synthesis and stimulates programmed cell death [16,17,18]. There are two main types of Stx, Stx1 and Stx2 with multiple subtypes in each group. Stx2a is most commonly associated with human infections [19].

The sequence diversity of Stx prophages affects Stx expression. The *stx* genes are located in the late region of the prophage, downstream of the antiterminator *Q* and upstream of the lysis cassette, and are controlled by the late promoter *p*R’ [20]. Protein Q binds to the Q utilization site (qut), which is found partially between the -10 and -35 sites of pR’, and allows the RNA polymerase to read through the terminator cassette [21]. The Q protein from lambda was unable to act as an antiterminator for the H-19B phage [22]. Sequence diversity of this region may thus affect the expression of *stx* [23,24]. Antiterminator *Q* affects *stx* expression, with *Q*_933_ in *E. coli* EDL933 related to higher *stx* expression [25], while its alleles, *Q*_21_ and *Q_O_*_111*:H-*_, which share a low amino acid identity with *Q*_933_, have different properties [24,26]. Genomic differences in the early transcription region also affect toxin production and phage induction. The sequence diversity of proteins O and P, which are in the early region, affect toxin expression [27].

Stx phages have a broad range of genome size ranging from 16 Kb to 68.7 Kb [28,29]. Such variation among the Stx genome, especially the late regulation region [26,30], may directly or indirectly change prophage induction and toxin production; however, sequence variation of the regulatory regions upstream of *stx* have not been linked to phage induction and *stx* expression. This study therefore aimed to determine the expression of *stx* under control of different *p*R’ regions in their native and non-native strains, demonstrating that the mosaic nature of stx phage affects their virulence and allows for the rapid evolution of Stx phages. Heterogeneous *p*R’ regions were retrieved from STEC differing in origin and sequence of the *stx* prophage. A DsRed based reporter system visualized *stx* expression and the interaction between different *p*R’ and different target regulatory systems were determined by cloning the reporter construct in different STEC. Previous studies have shown that when two lambdoid prophages are present in a cell both are induced; however, we found that this was not always the case [31].

## 2. Materials and Methods

### 2.1. Bacterial Strains and Culture Conditions

The STEC strains used in this study are listed in Table 1 [32]. Strain *E. coli* O104:H4 strain 11-3088 Δ*stx*::*gfp*::*amp^r^* was used as the reporter strain for DsRed expression; this strain is a derivative of the outbreak strain *E. coli* O104:H4 that was obtained by the replacement of *stx2a* by a *gfp::amp^r^* cassette [33]. *E. coli* were routinely grown in Luria-Bertani (LB) medium (BD, Fisher Scientific, Edmonton, CA, USA), at 37 °C with agitation at 200 rpm, or on LB agar plates with 1.5% agar (BD, Fisher Scientific). Ampicillin (50 g/L) and chloramphenicol (100 g/L) were added when required for plasmid maintenance.

### 2.2. Sequence Analysis and Phylogenetic Trees

For scaffolding the contigs and pairing, the contig(s) (Table 1) containing *stx* were retrieved and reference strains with a closed genome were determined by Nucleotide BLAST on the National Center for Biotechnology Information (NCBI) (https://blast.ncbi.nlm.nih.gov/Blast.cgi). To obtain the complete sequence of the target segment, reference genome sequences were downloaded from the NCBI nucleotide database and contigs were manually aligned with the references and assembled into a larger segment in Geneious (Biomatters, Auckland, New Zealand). Gaps between contigs were filled by Sanger sequencing.

Sequence alignment and phylogenetic analysis of the *p*R’ regions and *stx* genes were generated by Geneious. To generate the phylogenetic trees, sequences of the *p*R’ region were first aligned using MUSCLE [35]. Results of the alignment were used to build the tree. The *stx* from *Shigella dysenteriea* type 1 strain Sd197 (accession number: NC_007606) was included as the outgroup. Parameters “Tree build Method” and “Resampling Method” were set as “Neighbor-Joining” and “Bootstrap”, respectively, while the rest of the parameters were set to default values.

### 2.3. Nomenclature of Promotor Constructs

The *p*R’ region was determined as the region starting from the last 42 bp of the Q protein and ending by the first 39 bp of the *stx* to make sure that the *p*R’ from all candidate strains could be included. Plasmids containing the different *p*R’ were named as P*p*, followed by the strain number of the Food Microbiology culture collection at the University of Alberta (FUA number). For example, the pUC19 derived plasmid containing the *p*R’ fragment from *E. coli* was termed P*p*1302*.* Plasmids containing the *p*R’ region from strains with more than one *stx* gene were denoted by P*p*, followed by the FUA number and the abbreviation of the *stx* subtype. For example, the plasmids containing one of the two *p*R’ fragments from *E. coli* FUA1303 were denoted as P*p*1303-1 and P*p*1303-2a, respectively. Plasmids containing promotor regions from *E. coli* FUA1399, which harbors two *stx2a* genes, were denoted by the FUA number and the contig number, which were P*p*1399-28 and P*p*1399-79.

### 2.4. Construction and Validation of the pR’::rfp::chl^r^ Transcriptional Fusion Reporter System

To construct the *p*R’::*rfp*::*chl^r^* fusion reporter system, fragments *p*R’, *rfp*, and *chl^r^* were amplified from candidate STEC strains, plasmid pDsRed (Clontech, Mountain View, CA, USA), and plasmid pKD3 [36], respectively. Three fragments were ligated together and transformed into the vector pUC19. The plasmids and primers used are listed in Table 1 and Table 2.

Construct P*p*1302::*rfp*::*chl^r^* was transformed into *E. coli* O104:H4 11-3088 Δ*stx*::*gfp*::*amp^r^* and O157:H7 CO6CE900, respectively, to validate the RFP reporter system. To measure the phage induction level under the control of the same regulatory system, constructs P*rfp*::*chl^r^*, P*p*1302::*rfp*::*chl^r^*, P*p*1303-1::*rfp*::*chl^r^*, P*p*1303-2a::*rfp*::*chl^r^*, P*p*1306::*rfp*::*chl^r^*, P*p*1309-1c::*rfp*::*chl^r^*, P*p*1309-2d::*rfp*::*chl^r^*, and P*p*1311::*rfp*::*chl^r^* were transformed into *E. coli* O104:H4 11-3088 Δ*stx*::*gfp*::*amp^r^*. To determine the induction level in the native environment, constructs P*p*1303-1::*rfp*::*chl^r^*, P*p*1303-2a::*rfp*::*chl^r^*, P*p*1311::*rfp*::*chl^r^*, P*p*1399-28::*rfp*::*chl^r^*, and P*p*1399-79::*rfp*::*chl^r^* were transformed back into their parent strains: *E. coli* FUA1303, FUA1311, and FUA1399. To measure the induction level of the same prophage under the control of different regulatory system, P*p*1302::*rfp*::*chl^r^* was transformed into *E. coli* FUA1303, FUA1311, and FUA1399; P*rfp*::*chl^r^* was selected as the control. Electroporation transformation was employed to obtain the transformants.

To validate the fluorescence gene fusion reporter system, DsRed expression by strains harboring the reporter constructs was visualized by fluorescent microscopy under the Axio Imager microscope (Carl Zeiss Canada Ltd., Toronto, ON, Canada). Image acquisition was performed with multi-channel fluorescence imaging with filters for Rhodamine (red fluorescence) and GFP. Cells were grown in LB with a 0.5 µg/mL final concentration MMC (M0503-2MG, Millipore Sigma, St. Louis, MO, USA) for 4.5 h, and observed with a 10× or 40× objective lens and a 10× ocular. Pictures were captured by an AxioCam M1m 385 camera and viewed by Axio Vision software (v.4.8.2.0, Carl Zeiss Canada Ltd., Toronto, ON, Canada).

### 2.5. Determination of the Treatment Conditions for Flow Cytometry Detection

To prevent cell lysis prior to analysis by flow cytometry without interfering with the folding of DsRed, a time course experiment of heat inactivation was performed. The heating was performed at a time when DsRed was produced, but before the expression of phage genes resulted in cell lysis. Cells were induced with MMC (0.5 g/L) when OD_600_ reached 0.4~0.6 (exponential phase), further incubated for 3 h, and sampled every 30 min. Samples were heated to 60 °C for 5 min, resulting in cell inactivation but not cell lysis [37], and incubated at 4 °C for 27 h, 37 °C for 7 h, or 37 °C for 27 h.

**Figure 1:**
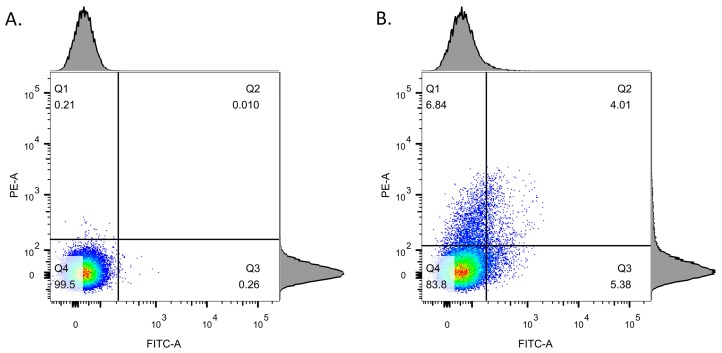
The gating strategy of *E. coli* O104:H4 11-3088 Δ*stx*::*gfp*::*amp^r^* (*p*1302::*rfp*::*chl^r^*) with or without MMC induction. (**A**) Dot plot of the negative control without MMC induction. (**B**) Dot plot of the sample induced with MMC for 4.5 h. Gating as represented by reference lines divided cell populations based on the fluorescent signal: Q1, RFP^+^, GFP^−^; Q2, RFP^+^, GFP^+^; Q3, RFP^−^, GFP^+^; Q4, RFP^−^, and GFP^−^. The gating was set to include 99.5% of the cells of the negative control.

A LSRFortessa™ X-20 cell analyzer (Biosciences, Mississauga, ON, Canada) was used to perform the cell analysis. Fluorescence was excited with a 488 nm Argon ion laser and followed by a 530/30–575/26 nm bandpass filters, and finally detected by side scatter detectors and a forward scatter detector. To adjust the detected cell number per second (e/s) between 300~3000 e/s, samples were resuspended and diluted between 1:100 and 20:100 with 1 mL 1× PBS (pH 7.4). Data was recorded by BD FACSDIVA^TM^ software (BD Biosciences, San Jose, CA, USA) and analyzed by FlowJo (BD Biosciences, San Jose, CA, USA) (Figure 1). The single cell population was defined by selecting the cell population located along the diagonal of the “FSC-A; FSC-H” dot plot, and “cells of favorite” was set as 100% of the singlets in the “FSC-A; SSC-A” dot plot. The gating strategy for the flow cytometric analysis is shown in Figure 1.

### 2.6. Flow Cytometry Detection of the Behavior of the pR’::rfp::chl^r^ Constructs in Different Target Strains

To evaluate the induction efficiency, exponential phase cultures were inducted by MMC (0.5 g/L), heat inactivated 4.5 h after induction, and measured by flow cytometer 27 h after induction (22.5 h after heating inactivation). The method used for the detection of the fluorescent cell population was the same as described above.

### 2.7. Statistical Analysis

The experiments were repeated at least three separate times (biological replicates). Statistical analysis was performed with SigmaPlot (v.12.5., Systat Software Inc., London, UK) using one-way analysis of variance (ANOVA). A *p*-value of ≤0.05 was considered statistically significant.

## 3. Results

### 3.1. Sequence Alignment and Phylogenetic Analysis

**Figure 2:**
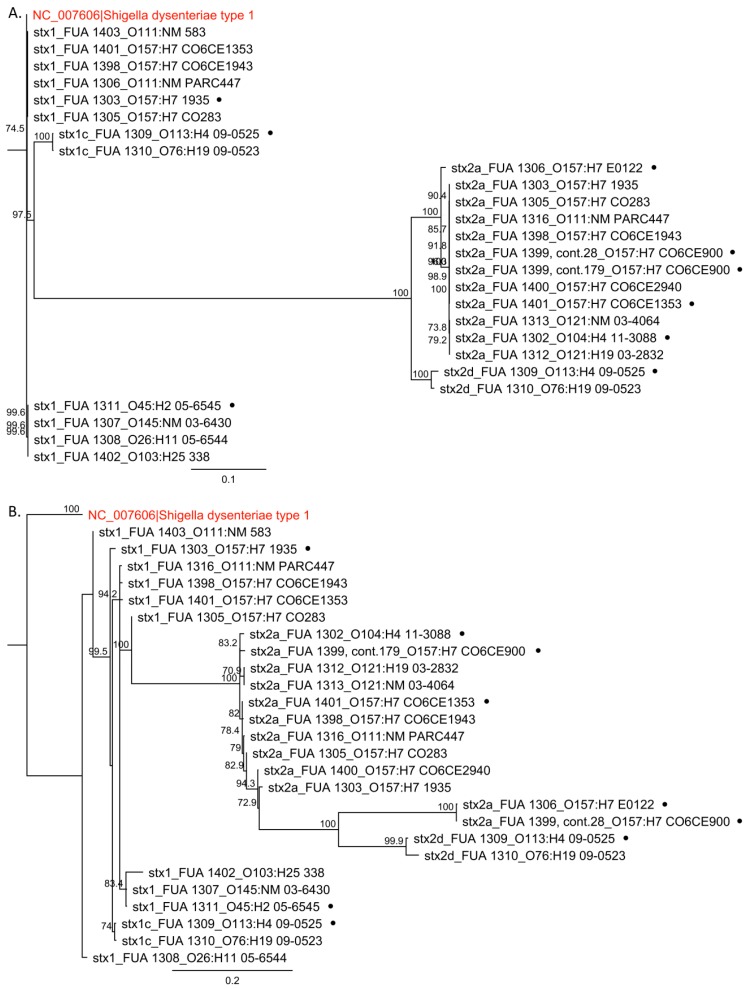
Phylogenetic tree analysis of the *stx* gene sequences and the DNA sequence of the corresponding *p*R’ fragments. The phylogenetic tree was based on 26 sequences from 17 STEC strains (Table 1). Neighbor-Joining trees were generated in Geneious using the Tamura–Nei model. The reliabilities of the internal branches were assessed using bootstrapping with 1000 pseudo-replicates. The scale bars represent the number of the substitution per site. Bootstrap values over 70% are displayed. *Shigella dysenteriea* type 1 strain Sd197 was included as the outgroup. Strains that had significant phylogenetic differences between the *p*R’ region and *stx* gene are highlighted by dots and were used in downstream studies. (**A**) Phylogenetic tree generated by comparing the *stx* genes, which included both subunit A and B. (**B**) Phylogenetic tree generated by aligning the *p*R’ region located between *Q* and *stx*.

Previous studies have demonstrated the mosaic nature of stx phages [30,38]. In this study, a phylogenetic analysis was performed to compare the *p*R’ region and *stx* to determine whether the phylogeny of *stx* corresponded to the phylogeny of the *p*R’ region that controls *stx* and prophage expression (Figure 2). The *stx* genes of the same subtype were located in the same clade (Figure 2A); *stx1* and *stx1c* were located in two separate clades where genes belonging to the *stx2* subtypes were all in the same branch. The phylogeny of *p*R’ regions was more heterogeneous (Figure 2B) and did not match the phylogeny of the corresponding *stx*.

**Figure 3:**
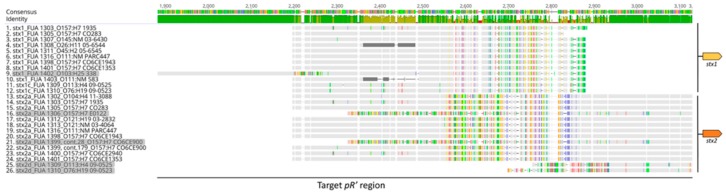
The sequence comparison of the *p*R’ regions. The toxin subtypes and the name of their host strains are listed on the left. Consensus is shown on top. Sequence identities are colored in green, yellow, and red, which indicate that the residue at that position is the same across all sequences, less than complete identity and very low identity, respectively. The schematic s*tx* genes were annotated behind the *p*R’ regions. The sequences that did not have the same *p*R’ site as the reference are shaded. The figure is provided in high resolution for large scale printing or viewing.

The late promoter region, which includes the *p*R’ promoter, is directly upstream of *stx* and downstream of *Q* [39]. To assess the sequence diversity, 26 sequences of the *p*R’ region were aligned (Figure 3). The comparison of the *p*R’ regions confirmed that the sequences of *p*R’ regions were highly divergent even if they regulated the same *stx* subtype (Figure 3). Most of the sequence differences in the *p*R’ regions were caused by single nucleotide changes and not the insertion of a whole flanking region, which suggested the possibility of functional diversity during phage induction [40]. Several *p*R’ regions including *p*1402, *p*1309-2d, *p*1310-2d, *p*1306, and *p*1399-28 lacked the *p*R’ site that was identified in highly virulent strains (Acc. No. AP000400) [41]. In order to determine the effect of the pR’ region on phage induction levels, we selected nine prophages with diverse sequences of *stx* and the *p*R’ region for subsequent analyses excluding closely related sequences.

### 3.2. Construction and Validation of the pR’::rfp::chl^r^ Transcriptional Fusion

**Figure 4:**
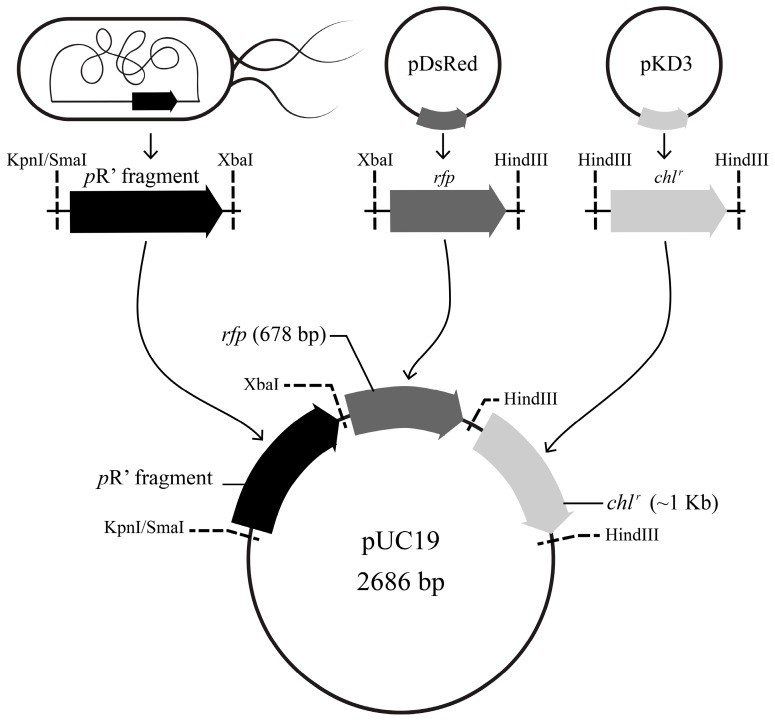
Scheme representing the construction of P*p*R’::*rfp*::*chl^r^* reporter plasmids. Arrows with direction indicate the transcription orientation. The black arrow represents the *p*R’ region; dark gray is the *rfp* fragment; light gray is the chloramphenicol resistance gene. Dashed lines indicate restriction sites; note that *p*1402 used restriction enzymes SmaI/XbaI, since the sequence of *p*1402 contains the restriction site KpnI. The fragment of the *p*R’ region and *rfp* were transformed into pUC19 vector, followed by a *chl^r^* fragment for positive screening.

To determine the role of the *p*R’ region in *stx* expression, we amplified the *p*R’ fragments from 16 strains by PCR and ligated the *p*R’ fragments into the plasmid pUC19, respectively. The DsRed reporter protein and the antibiotic resistance gene *chl^r^* was introduced into the vector, downstream of the *p*R’ region. The resulting plasmid is depicted in Figure 4 (schematic rings).

**Figure 5:**
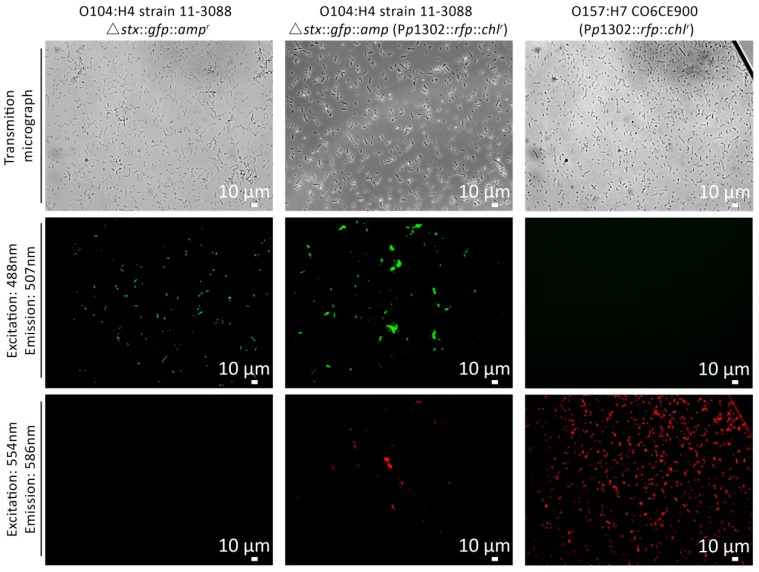
Microscopic observation of strains of *E. coli* expressing GFP or DsRed under control of Shiga-toxin promotors after MMC induction. Cells were visualized at 400× magnification by light microscopy or fluorescence microscopy as indicated. Shown from left to right are *E. coli* O104:H4 11-3088 Δ*stx*::*gfp*::*amp^r^* (negative control for DsRed expression); *E. coli* O104:H4 11-3088 Δ*stx*::*gfp*::*amp^r^* (P*p*1302*::rfp::chl^r^*), and *E. coli* O157:H7 CO6CE900 (P*p*1302*::rfp::chl^r^*) (negative control for GFP expression). MMC induction was performed 4.5 h before microscopy observation.

To validate the *p*R’::*rfp*::*chl^r^* transcriptional fusion, *E. coli* O104:H4 11-3088 Δ*stx*::*gfp*::*amp^r^* (P*p*1302::*rfp*::*chl^r^*) and *E. coli* O157:H7 CO6CE900 (P*p*1302::*rfp*::*chl^r^*) were induced by 0.5 g/L MMC for 4.5 h (Figure 5). *E. coli* O104:H4 11-3088 Δ*stx*::*gfp*::*amp^r^* was used as the negative control. In this strain, *stx* was replaced by *gfp* to visualize protein expression by fluorescence microscopy or flow cytometry [33]. In the absence of the *p*R’ construct, only GFP positives could be observed after induction, whereas RFP positives were only detected in the target strain carrying a *p*R’::*rfp* construct. Moreover, *E. coli* O104:H4 11-3088 Δ*stx*::*gfp*::*amp^r^* (P*p*1302::*rfp*::*chl^r^*) showed both GFP and RFP positive cells, which demonstrated that the expression of the chromosomal *gfp* and the plasmid *rfp* were not affected by each other (*p* ≥ 0.05).

### 3.3. Detection of Stx Induction Levels in STEC Populations

Since *stx* is located in the late lytic region [42], Stx induction also induces the lytic cycle and eventually results in cell lysis, which obscures the detection of cells by flow cytometry. Thus, cultures were inactivated with heat 4.5 h after MMC induction, followed by incubated at 37 °C for 22.5 h. This protocol enabled the quantification of the proportion of cells expressing GFP or DsRed, or both, by flow cytometry (Figure 1).

**Figure 6:**
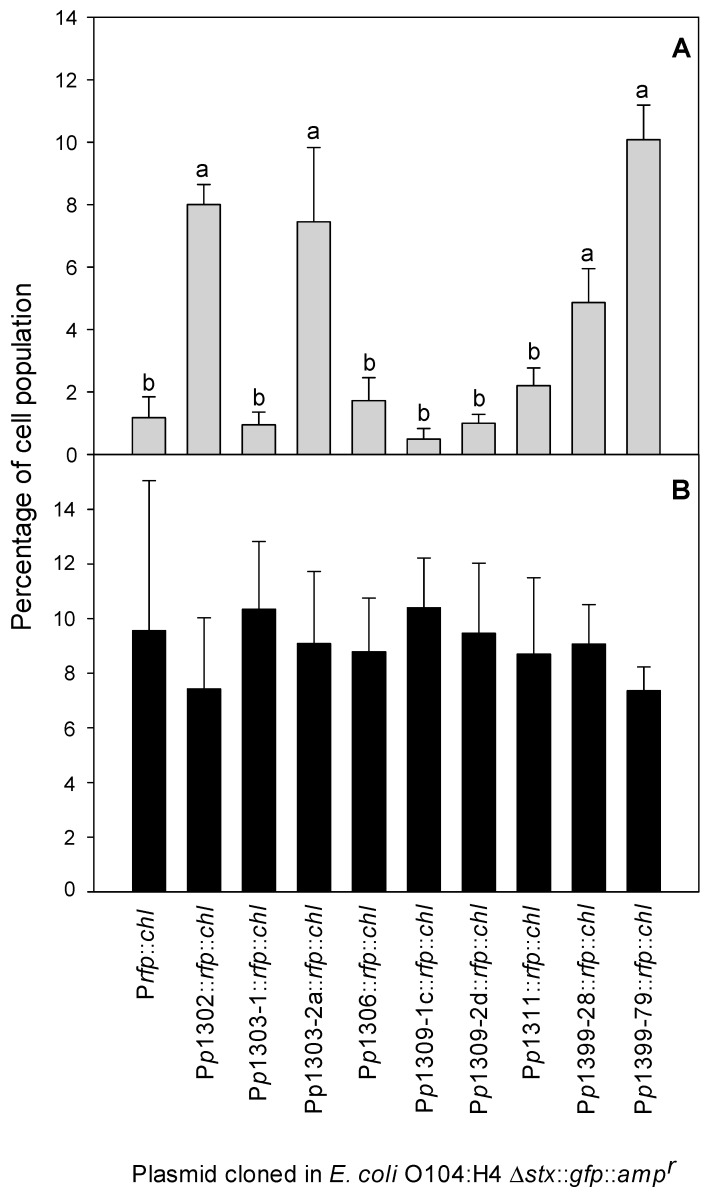
Expression of GFP and DsRed by *E. coli* O104:H4 11-3088 Δ*stx*::*gfp*::*amp^r^* (P*p*R’::*rfp*::*chl^r^*) transformants after MMC induction. (**A**) Percentage of the population expressing plasmid-encoded DsRed under control of the plasmid-encoded promotor indicated. The promotorless plasmid P*rfp*::*chl^r^* served as the negative control. (**B**) Percentage of the population expressing the chromosomal gene coding for GFP under control of the native promotor. The percentage of the red or green fluorescent cell population was determined by flow cytometric analysis and is shown as mean ± standard deviations of quadruplicate independent experiments. Bars that do not share a common letter are significantly different (*p* ≤ 0.05).

To determine the impact of the diversity of the *p*R’ region, we selected 16 transformants that represented various combinations of the *p*R’ and regulatory regions, and measured the induction levels in the presence and absence of the MMC with flow cytometry. Initially, we measured the induction level in seven *E. coli* O104:H4 11-3088 Δ*stx*::*gfp*::*amp^r^* (P*p*R’::*rfp*::*chl^r^*) transformants. Under the control of regulatory proteins of the *E. coli* O104:H4 11-3088 prophage, transformants carrying the constructs *p*1302::*rfp*::*chl^r^*, *p*1303-2a::*rfp*::*chl^r^*, *p*1399-28::*rfp*::*chl^r^*, and *p*1399-79::*rfp*::*chl^r^* showed higher DsRed expression; other transformants did not express DsRed (Figure 6A). GFP expression among the transformants was not different (Figure 6B) (*p* ≥ 0.05), indicating that expression of the chromosomal *gfp* was not influenced by the plasmid-encoded heterologous *p*R’ region.

**Figure 7:**
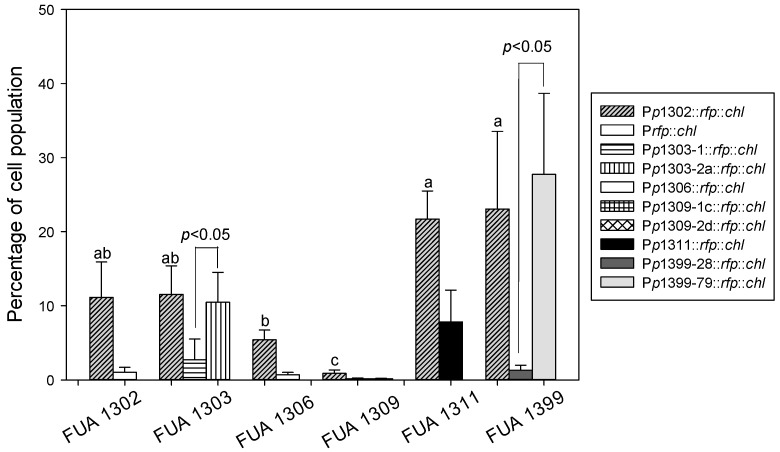
Percentage of the population of strains of *E. coli* expressing DsRed under the control of different Shiga-toxin regulatory sequences. To determine the effect of the native regulator to the *p*R’ region, the *p*R’::*rfp*::*chl^r^* constructs were cloned from the target strains and transformed back into their parent strains. To determine whether the same *p*R’ region was differentially expressed in different strains, the construct *p*1302::*rfp*::*chl^r^* was transformed into all target strains and its parent strain *E. coli* FUA1302 O104:H4. Transformants were induced with MMC. Bars are grouped by the six target strains, the bars represent different *p*R’ constructs shown in the figure legend. Bars with the same pattern that do not share a common letter differed significantly. The percentage of fluorescent cells are shown as mean ± standard deviations of quadruplicate independent experiments (*p* ≤ 0.05).

To investigate the behavior of the *p*R’ region under the control of its parent prophage, we measured the induction level of eight transformants: *E. coli* FUA1303 (P*p*1303-1::*rfp*::*chl^r^*), *E. coli* FUA1303 (P*p*1303-2a::*rfp*::*chl^r^*), *E. coli* FUA1306 (P*p*1306::*rfp*::*chl^r^*), *E. coli* FUA1309 (P*p*1309-1c::*rfp*::*chl^r^*) and *E. coli* FUA1309 (P*p*1309-2d::*rfp*::*chl^r^*), *E. coli* FUA1311 (P*p*1311::*rfp*::*chl^r^*), *E. coli* FUA1399 (P*p*1399-28::*rfp*::*chl^r^*), and *E. coli* FUA1399 (P*p*1399-79::*rfp*::*chl^r^*) (Figure 7). To determine the induction behavior resulting from the combination of the same *p*R’ and different regulatory regions, we transformed *p*1302::*rfp*::*chl^r^* into six different strains (Figure 7). We examined the induction levels in *E. coli* FUA1303, *E. coli* FUA1309, and *E. coli* FUA1399, which carry two prophages in their chromosome. The percentage of RFP positives revealed that not all of the prophages can be induced by MMC: P*p*1303-1::*rfp*::*chl^r^* and P*p*1399-28::*rfp*::*chl^r^* were not induced; in *E. coli* FUA 1309, both P*p*1309-1c::*rfp*::*chl^r^* and P*p*1309-2d::*rfp*::*chl^r^* were uninduced. We also compared the induction level of the *p*1302::*rfp*::*chl^r^* in different STECs and found significant differences among the six transformants. The pR’ promoter region from 1302 was regulated differently by different strains, in *E. coli* FUA1303, *E. coli* FUA1311, and *E. coli* FUA1399, the induction level of P*p*1302::*rfp*::*chl^r^* was comparable to its native strain; while in *E. coli* FUA 1309, the expression was lower (*p* ≤ 0.05). Additionally, the percentage of fluorescent cells in *E. coli* FUA1306 and *E. coli* FUA1311 with the heterologous promoter P*p*1302::*rfp*::*chl^r^* was higher than the expression of the same protein under control of the homologous promoter in *E. coli* FUA1306 (P*p*1306::*rfp*::*chl^r^*) and *E. coli* FUA1311 (P*p*1311::*rfp*::*chl^r^*) (*p* ≤ 0.05). Finally, the induction levels among P*p*1302::*rfp*::*chl^r^*, P*p*1309-1c::*rfp*::*chl^r^*, and P*p*1309-2d::*rfp*::*chl^r^* were not different when under the control of the prophages from *E. coli* FUA 1309 (*p* ≥ 0.05). Taken together, these data demonstrate that the sequence diversity of *p*R’ as well as prophage-encoded regulatory proteins resulted in a concomitant diversity of expression levels.

## 4. Discussion

STEC genomes have a high degree of sequence diversity [26,43,44,45] and different STECs differ in their virulence with disease symptoms ranging from mild diarrhea to hemolytic-uremic syndrome leading to death [44]. Sequence diversity in the early regulatory region directly affects *stx* expression and toxin production [46,47,48], and accounts for differences in virulence. The present study provides evidence that sequence diversity in the late promoter region also contributes to different Stx expression in STEC. As Stx prophages not only confer virulence to STEC, but also convert commensal *E. coli* to pathogens [49,50], differences in the expression of late phage genes likely results in different degrees of virulence of different strains.

Sequence analysis of the *p*R’ region revealed the presence of a great number of nucleotide differences. Of the two promoters upstream of *stx*, the distal promoter *p*R’ controls Stx production [20]. To investigate the genetic relationship between *p*R’ and *stx*, we conducted a phylogenetic analysis for these two sequences. The *stx* were highly conserved within the *stx* subtypes, whereas the *p*R’ regions, whose *stx* are from the same subtype, are distinct from each other (Figure 3). This is in agreement with previous studies where the late gene region of Shiga phages exhibits considerable genetic diversity [30,42] and the emergence of the STECs in *E. coli* cannot be predicted through the serotypes [51].

Induction efficiency is positively correlated to Stx production and pathogenicity [44,52,53]. To determine the effect of the diversity in the late promoter region on the behavior of STECs, we transformed *p*R’::*rfp*::*chl^r^* constructs with representative promoter sequence structures into different target strains and quantified gene expression with fluorescent reporter proteins. Bacterial behavior is commonly assessed in bulk [51,52]. To include the stochastic switching during detection [54], we employed flow cytometry to allow the efficient measurement at a single-cell level [33,34]. As one of the most commonly used inducers, MMC was chosen to induce cultures in this research. However, lambdoid phages show different induction efficiency in response to different induction agents [52]. Thus, it is possible that the efficiency of induction may change under the treatment of other induction agents.

The use of *p*R’ from seven different Stx prophages to control DsRed expression in *E. coli* O104:H4 11-3088 Δ*stx*::*gfp*::*amp^r^* demonstrated that the sequence diversity of the *p*R’ region corresponded to different levels of gene expression. *E. coli* O157:H7 harboring *stx2* under the control of *Q_21_* rather than *Q_933_* may exhibit a Stx2-negative phenotype [55]. The present study confirmed that prophage encoded regulatory proteins impact Stx expression as the same construct showed different expression levels in different strains. However, prophages in *E. coli* FUA1302 and *E. coli* FUA1311 both harbored the typical *p*R’ site [41] and the highly conserved *Q_933_* [23]. Induction efficiencies of P*p*1302::*rfp*::*chl^r^* and P*p*1311::*rfp*::*chl^r^* were different under the control of the *E. coli* FUA1302 prophage. We thus propose that the *Q* and *p*R’ sites are not the only determinants of induction efficiency of the late transcript region; sequence diversity in the late promoter region *p*R’ [26] also regulates induction efficiency. Moreover, the similar GFP populations among samples indicates that the expression of the plasmid *rfp* did not interrupt the regulation of the chromosomal *gfp*.

A sequence of the *p*R’ site is related to high Stx production. We thus used this reported *p*R’ site as our reference to investigate our candidate *p*R’ sites. The reference *p*R’ site (accession number: AP000400) [41], which is related to high Stx production [27,40], was not found in the candidate prophages from *E. coli* FUA 1306, *E. coli* FUA 1309, and *E. coli* FUA 1399; and the constructs that do not have the *p*R’ site as the reference did not express DsRed after induction. Additionally, it seems that different types of *p*R’ sites randomly combine with different *stx* genotypes: P*p*1399-28::*rfp*::*chl^r^* has the same *stx2a* as P*p*1399-79::*rfp*::*chl^r^*, but different *p*R’ sites. Another finding is that the induction level of P*p*1303-1::*rfp*::*chl^r^*, which harbors the same *p*R’ site as the reference sequence, did not increase significantly. Typically, strains with the reference *p*R’ site have a higher expression level; this phenotype might relate to the change of the binding ability of RNA polymerase to the prophage DNA and Q [56], and thus affect phage metabolism and physical behavior during lysis.

The presence of two more *stx* prophages was proposed to increase the pathogenicity of the STEC by changing the toxin expression [57]. However, other research has reported that lysogens with more than one phage produce less toxin [58]. In this study, *E. coli* FUA1399, prophages 1399-28 and 1399-79 carry the same *stx2a*, which is related to a high rate of HUS [59]. While P*p*1399-79::*rfp*::*chl^r^* was highly induced, P*p*1399-28::*rfp*::*chl^r^* was not induced. This indicates that expression of the Shiga toxin in a STEC is not determined by the number of Stx prophages, but by the expression levels that are controlled by the interaction of the regulatory Q protein(s) and the *p*R’ site.

Genetic exchange through phages generates genomic diversity and promotes the evolution of the host bacteria. Such gene transfer helps bacteria survive in the diverse environments in nature, but also gives the chance for bacteria to gain virulence determinants from pathogenic strains, thus generating new pathogens [3,7,45,60,61]. As a food-borne pathogen, *E. coli* gaining *stx* during evolution has a substantial impact on human health. Beef cattle are a main source of STEC transmission to humans, either directly through the meat supply or indirectly through contamination of water and plant foods [62,63]. Predatory protists are proposed to exert a selective pressure for maintenance of the Shiga-toxin prophage by commensal *E. coli* in ruminants [7]. It is tempting to speculate that the sequence diversity of Shiga-toxin prophages responds to the diversity of predatory protozoa in the gut microbiome of ruminants [64]. Understanding the link between genomic diversity of Stx prophages and Stx production may provide solutions to predict and prevent STEC contamination in ruminants and human STEC infections.

## 5. Conclusions

In this study, the phylogenetic relationship of the *stx* confirmed previous investigations that the sequence structure of *stx* is highly conserved. However, the phylogenetic analysis of the *p*R’ region revealed that this late promoter region was more heterogeneous. The combination of the fluorescent reporter fusion system and flow cytometric analysis confirmed that toxin expression could be observed at the single-cell level. Our data from the phylogenetic analysis and the determination of toxin expression levels of the *p*R’::*rfp*::*chl^r^* transformants indicated a correlation between the diversity of the late promoter *p*R’ region and the efficiency of toxin expression. These results may provide evidence that in addition to the diversity of the functional genes, the diversity of the late promoter region, *p*R’ region also contributes to the level of toxin expression.

## Figures and Tables

**Table 1:** Strains and plasmids used in this study.

Accession Numbers	Strains and Serotype	FUA Number Used for Plasmid Nomenclature	Description	Ref.
LDYN00000000	*E. coli* O26:H11 05-6544	1308	*stx1*	[32]
LDZZ00000000	*E. coli* O121:H19 03-2832	1312	*stx2a*	[32]
LEAA00000000	*E. coli* O121:NM 03-4064	1313	*stx2a*	[32]
LEAB00000000	*E. coli* O145:NM 03-6430	1307	*stx1*	[32]
LEAD00000000	*E. coli* O157:H7 1935	1303	*stx1 stx2a*	[32]
LEAE00000000	*E. coli* O157:H7 CO6CE900	1399	*stx2a*	[32]
LEAF00000000	*E. coli* O157:H7 CO6CE1353	1401	*stx1 stx2a*	[32]
LEAG00000000	*E. coli* O157:H7 CO6CE1943	1398	*stx1 stx2a*	[32]
LEAH00000000	*E. coli* O157:H7 CO6CE2940	1400	*stx2a*	[32]
LEAI00000000	*E. coli* O157:H7 CO283	1305	*stx1 stx2a*	[32]
LEAJ00000000	*E. coli* O157:H7 E0122	1306	*stx2a*	[32]
LECF00000000	*E. coli* O103:H25 338	1402	*stx1*	[32]
LECH00000000	*E. coli* O104:H4 11-3088	1302	*stx2a*	[32]
LECI00000000	*E. coli* O111:NM 583	1403	*stx1*	[32]
LECJ00000000	*E. coli* O111:NM PARC447	1316	*stx1 stx2*	[32]
LECK00000000	*E. coli* O113:H4 09-0525	1309	*stx1c stx2d*	[32]
LECM00000000	*E. coli* O45:H2 05-6545	1311	*stx1*	[32]
LECN00000000	*E. coli* O76:H19 09-0523	1310	*stx1c stx2d*	[32]
	*E. coli* DH5α			
	*E. coli* Top10		pUC19	
	*E. coli* Top10		pRFP	
	*E. coli O104:H4 11-3088 Δstx::gfp::amp*		*stx* gene replaced with *gfp*	[34]

**Table 2:** Primers used for obtaining *p*R’ and *rfp* fragments.

Primer	Sequence (5′-3′) ^a)^	Restriction Site
LP F1-1	5′-CGGGAAGGTACCACCTCTGTATTTTATCAG-3′	KpnI
LP R1-3	5′-GGGCCGTCTAGAAAAGAAAAAAGTTAGCAC-3′	XbaI
LP F2-2	5′-ATTAGTCCCGGGCTTGGATTTATTGATGGT-3′	SmaI
LP R3-2	5′-ATAACGTCTAGATAACAGGCACAGTACCCA-3′	XbaI
LP F3-2	5′-AGCGGTACCAAAAACCGGAAACGTGTA-3′	KpnI
LP F4-1	5′-TGCGTAGGTACCAGCGTCTATAATTGTATG-3′	KpnI
LP R4-2	5′-GCATTATCTAGACAACAGGCACAGTATCCA-3′	XbaI
RFP F-2	5′-CTGATATCTAGAATGGCCTCCTCCGAG-3′	XbaI
RFP R-5	5′-ATCTGTAAGCTTCTACAGGAACAGGTGGT-3′	HindIII

^a)^ Restriction enzyme sites are underlined.
